# Psychometric properties of the Iranian version of the Copenhagen Burnout Inventory 

**DOI:** 10.15171/hpp.2019.19

**Published:** 2019-05-25

**Authors:** Elham Javanshir, Iman Dianat, Mohammad Asghari-Jafarabadi

**Affiliations:** ^1^Department of Occupational Health and Ergonomics, Tabriz University of Medical Sciences, Tabriz, Iran; ^2^Road Traffic Injury Research Center, Tabriz University of Medical Sciences, Tabriz, Iran; ^3^Department of Statistics and Epidemiology, Tabriz University of Medical Sciences, Tabriz, Iran

**Keywords:** Burnout, Iran, Psychometrics

## Abstract

**Background:** The Copenhagen Burnout Inventory (CBI) is a commonly used tool for evaluation of job burnout in three (personal, work-related and client-related) domains. The aims of this study were to translate and investigate the psychometric properties of the Iranian (Persian) CBI.

**Methods:** A total of 750 employees of different occupations (from educational centres, healthcare, industrial settings, and social services) participated in this descriptive methodological study. A forward-backward procedure was applied and content validity was evaluated by a panel of10 experts. Exploratory and confirmatory factor analyses were used for construct validity. The internal consistency (using Cronbach’s alpha), test-retest reliability (using intraclass correlation coefficient – ICC), and feasibility (using ceiling and floor effect) were also assessed for this tool.

**Results:** Content validity of the Persian CBI was established. Three-factor structure of the PersianCBI was supported by the factor analysis, and this confirmed the construct validity of the instrument. The internal consistency (Cronbach’s alpha ranged from 0.82 to 0.90) and test-retest reliability (ICC ranged from 0.85 to 0.95) were excellent and there was no ceiling or floor effect.

**Conclusion: ** The Persian CBI is a valid and reliable measurement tool for burnout in the Iranian context.

## Introduction

Occupational burnout has many negative consequences in family, social and individual life as well as in organizations and work environments, and is a key factor associated with absenteeism, job cracks, sequential delays, various complaints, job changes, and interpersonal conflicts with colleagues.^1-3^ Burnout has different definitions, but the most commonly used is “a state of physical, emotional, or mental exhaustion caused by long-term involvement in situations that are emotionally demanding”.^1^The concept of burnout was introduced in the psychosocial literature during the 1970s. Freudenberger^3^ and Maslach & Jackson^4^ were two investigators who independently introduced this concept. According to Maslach and Jackson: “burnout is a syndrome of emotional exhaustion, depersonalization, and reduced personal accomplishment that can occur among individuals who do ‘people work’ of some kind”. However, according to this definition, burnout is limited to human service work and its associated factors (e.g., high emotional load).^2^ Burnout syndrome was initially introduced for the service professions (e.g., healthcare workers, teachers, etc) and has been generally evaluated through Maslach Burnout Inventory (MBI).^5^ The MBI has been developed to assess burnout syndrome based on three consequences: emotional exhaustion, depersonalization, and lack of personal fulfilment.^6,7^ However, such a condition is only associated with stressful working conditions independent of the relationship with other people.^3^

Fatigue and emotional exhaustion seems to be the main concepts of burnout.^1-7^ The Danish National Institute of Occupational Health identified constraints in the use of MBI for evaluation of burnout.^8^ This institute reviewed the literature and conducted a pilot test using MBI, and finally developed a new instrument, namely Copenhagen Burnout Inventory (CBI), which allows measuring burnout in different settings (not just the service professions) with higher accuracy than MBI.^2^ The new tool overcomes the limitations of MBI and satisfies the need to measure burnout suitably.^2^ The hypothesis for developing CBI was that burnout syndrome is a phenomenon that is characterized by a core of exhaustion (both physical and psychological).^9^ This exhaustion develops across different life domains (e.g., personal sphere, work experience and interaction with clients), and these domains correspond to the three subscales that constitute the CBI (e.g., personal burnout, work-related burnout, and client-related burnout).^2^

So far, several studies have been conducted on the validity and reliability of the CBI in different countries. Psychometric evaluation of the CBI in Spain among four different occupational groups (teachers, healthcare workers, industry workers, and social service staff) showed that this tool is a reliable tool for measuring occupational burnout.^10^ Other studies in China, South Africa, New Zealand, Portugal, Brazil, Italy and Malaysia have also shown the validity and reliability of this tool.^11-17^ Nevertheless, the validity and reliability of the CBI in other languages and cultures has yet to be determined. This is the case for Iranian language. It should be noted that although a recent study has been conducted on the psychometric properties of this tool in Iran, it only considers nursing population (e.g., service professions).^18^ Therefore, additional studies seem to be necessary to characterize the psychometric properties of this tool for other occupational groups, particularly for industrial workers. Therefore, the aim of this paper was to examine the acceptability, reliability and construction validity of the Iranian version of three CBI scales in workers of different occupations.

## Materials and Methods

### Study design, setting, and participants

This descriptive methodological study was conducted during a 6-month period (January to June, 2018) in the city of Tabriz–Iran. The study population consisted of workers within four organizations of different types: educational areas (administrative staff, teachers, support staff), social work centres (residential and non-residential), healthcare centres (a primary care unit and a group of hospital residents) and workers within the industry sector. A total of 750 participants were selected using a multistage stratified random sampling technique. The number of participants from educational areas, social work centres, healthcare centres, and industry sector were 189, 190, 187, and 184, respectively. Being a full***-*** time employee with at least 1-year job tenure and having no chronic mental/physical problem (determined by self-report) were considered as inclusion criteria for the study. Data were collected using the Iranian version of the CBI. Demographic details of the study participants (age, gender, and educational level) were also recorded.

### Copenhagen Burnout Inventory

The CBI was developed by Kristensen et al during the Danish longitudinal study of burnout among employees in the human service sector.^2^ The CBI is a 19-item tool for measuring burnout in three domains including personal (6 items), work-related (7 items), and client-related domains (6 items). The personal burnout has six questions (questions 1–6), which are related to prolonged physical and psychological exhaustion. The work-related burnout has seven questions (question 7–13), which are associated with the long-term physical and psychological exhaustion in an individual due to his/her work. The client-related burnout has six questions (questions 14–19) which are related to the long-term physical and psychological exhaustion due to the individual’s work with clients.

For the personal burnout, each item has a 5-point Likert scale format as: “Always”, “Often”, “Sometimes”, “Seldom”, and “Never/almost never”. If the participant answers less than three questions, then the respondent is classified as non-responder. Item scoring is as follows: Always = 100, Often = 75, Sometimes = 50, Seldom = 25, and Never/almost never = 0. Total score for this scale is calculated as the average of the scores on the items. Therefore, the total score ranges from 0 to 100, with the lowest score indicating the desired and the highest score indicating an undesirable situation.

For the work-related burnout, there are two answer formats. The response format for first three questions is as: “To a very high degree”, “To a high degree”, “Somewhat”, “To a low degree”, and “To a very low degree”. The response format for last four questions is as: “Always”, “Often”, “Sometimes”, “Seldom”, and “Never/almost never”. If less than four questions have been answered by the respondent, it is classified as non-responder. The scoring system for this scale is the same as for the first scale.

There are also two response formats for the client-related burnout. The response format for first four questions is as: “To a very high degree”, “To a high degree”, “Somewhat”, “To a low degree”, and “To a very low degree”. The response format for last two questions is as: “Always”, “Often”, “Sometimes”, “Seldom”, and “Never/almost never”. If less than three questions have been answered by the participant, it is classified as non-responder. The scoring system for this scale is the same as for the two previous scales.

The CBI was converted into Persian (Iranian language) using a forward–backward translation process. The for­ward translation was performed by two specialists in the field of psy­chology. The back translation was performed by two specialists in the field of language. The English back–translation was then reviewed and checked for clarity and wording. The final questionnaire was revised based on the feedback from a sample of 30 participants through a pilot study.

With regard to qualitative evaluation, the questionnaire was re­viewed for content validity by an expert panel of 10 specialists in the fields of psy­chologists, ergonomists, and occupational health. In addition, two sets of questions (based on 4-point scale response format) were delivered to the expert panel members for quantitative evaluation. One set included questions regarding relevancy, clarity and simplicity of the items (for calculation of content validity index – CVI) and another set was related to the necessity of each item (for calculation of content validity ratio – CVR). CVI and CVR values > 0.79 and 0.62, respectively, were con­sidered appropriate considering the number of expert panel members.^19^

### Statistical analysis 

Statistical analysis was carried out with SPSS 21.0 (IBM Inc., Armonk, NY, USA) and AMOS 18.* P* values less than 0.05 were considered statistically significant. Stability reliability and internal consistency of the scale (performed on a sample of 30 subjects during a two-week interval) were evaluated using intraclass correlation coeffi­cient (ICC) and Cronbach’s α, respectively. Considering the nature of the analysis, two-way mixed, consistency and an average measure ICC was used. For both the stability reliability and internal consistency, values ≥0.7 was considered good.^20^ Ceiling and floor effects were evaluated using percentage of scores at the boundaries of the scaling.^21^ Structure of the measure was assessed by exploratory factor analysis (EFA) using principal axis factoring extraction procedure and direct oblimin rotation with Kaiser normalization. The number of extracted factors was determined using the scree plot method. Bartlett’s test of sphericity, Kaiser-Meyer-Olkin (KMO) measure of sampling adequacy and total variance explained were used to assess model sufficiency.^22^ KMO values higher than 0.7, significant values of the Bartlett’s test of sphericity (<0.05), and factor loadings ≥0.3 were considered for interpretation.^23^ Confirmatory factor analysis (CFA) was applied to assess the fit between EFA extracted model and observed data (asymptomatic covariance matrix = weighted matrix; input matrix = covariance matrix). The fit of the model was evaluated using various fit indices including root mean square error of approximation (RMSEA) (< 0.08), χ2 / df (< 5), adjusted goodness of fit index (AGFI) (> 0.9), goodness of fit index (GFI), and comparative fit index (CFI).

## Results

### Sample characteristics

A total of 750 subjects participated in the study (438 males, 58.4%; 312 females, 41.6%). The missing items ranged between 0.13% and 0.67%, which were deleted list wise. The age of participations ranged from 20 to 61 years (mean = 45.3 years; SD = 5.2 years). The majority of participants were married (n = 604, 80.5%). Among them, 11.7% (88) had primary school education, 2.4% (18) had secondary school education, 24.6% (185) had diploma, 19.5% (146) had undergraduate degree, and 41.8% (313) had postgraduate degree. In terms of the occupation, 189 (25.2%) were teachers, 186 (24.8%) were healthcare employees, 184 (24.5%) were industrial employees, and 190 (25.3%) were in social services.

### Content validity

The scores of CVI and CVR of the Persian version of CBI are presented in Table 1. According to these results, CVI ranged between 0.91 and 1.00, and CVR ranged be­tween 0.85 and 1.00, which indicates satisfactory results for each item and also for the Persian version of CBI.

### Construct validity 

*Exploratory factor analysis*

The results showed that the KMO measure of sampling accuracy was 0.941, which justifies the sufficiency of the model. The results of Bartlett’s test of sphericity (χ^2^(750) = 7821.185; *P* < 0.000) was also in agreement with the KMOs.^22^ Three factors were obtained from the factor analysis as follows:

- Factor 1: feel tired (item: PB_1_), physically exhausted (item: PB_2_), feel weak and susceptible to illness (item: PB_6_), emotionally exhausted (item: PB_3_), feel worn out (item: PB_5_), and cannot take it anymore (item: PB_4_)
- Factor 2: energy to work with clients (item: CB_3_), find it hard to work with clients (item: CB_1_), frustrating to work with clients (item: CB_2_), tired working with clients (item: CB_5_), give more than get back (item: CB_4_), and able to continue working with clients (item: CB_6_)
- Factor 3: work frustrated (item: WB_3_), feel burnt out (item: WB_2_), feel worn out at the end of the work (item: WB_4_), emotionally exhausting (item: WB_4_), feel that every working hour is tiring (item: WB_6_), and exhausted in the morning (item: WB_5_).

The total variance explained was determined to be 62.96% (Factor 1 = 47.59%; Factor 2 = 9.64%, Factor 3 = 5.71%). One item (item: WB_7_) with low communalities (<0.2) was deleted from the analysis, and therefore the results were revised after deleting this item. Factors and factor loading for each test item are presented in Table 2. It can be seen from this table that cut-off values are >0.3 for factor loadings, suggesting that all items strongly loaded on the Iranian version of CBI. One deleted item (item: WB_7_) had small value in loadings. Moreover, factors were correlated, which justifies the use of direct oblimin rotation method (corr >0.3 among factors).

### Ceiling and floor effects 

The Persian version of the CBI showed no ceiling or floor effects. The results of ceiling and floor effect are presented in Table 3.

*Confirmatory factor analysis*

**Figure 1:**
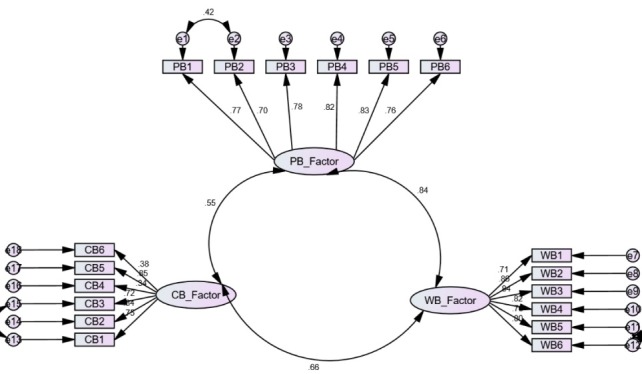
Relations between items and factors and between factors (from confirmatory factor analysis). All relations between factors and items as well as between the factors were significant (*P* < 0.05).

According to the CFA analysis, the model fit was confirmed by the indices: χ^2^/df = 4.38 <5; SRMR = 0.054 < 0.1; RMSEA = 0.067 <0.08 and 90% HI: 0.073; CFI = 0.95 >0.90; NFI = 0.93 >0.90; GFI = 0.917 >0.91; AGFI = 0.91 > 0.90; RFI = 0.92 >0.9; and IFI = 0.95 <1.^20,24,25^ Evaluation of the relationships between parameters and factors based on this model revealed that the items had significant loadings on the three factor solution (standardized factor loadings ranged between 0.34 and 0.86, as shown in Figure 1).

Moreover, correlations between factors were as follows: factor 1 and factor 2 (*r* = 0.756; *P* < 0.001); factor 1 and factor 3 (*r* = 0.519; *P* < 0.001); and factor 2 and factor 3 (*r* = 0.641; *P* = 0.001). The findings indicate that the EFA and CFA analyses confirm the models, and consequently the construct validity of this tool.

### Reliability 

Internal consistency reliability of the Persian CBI (evaluated using Cron­bach’s α coefficient) was 0.90, 0.82, and 0.88 for Factor 1, Factor 2, and Factor 3, respective­ly, which is satisfactory. Test-retest reliability (evalu­ated by ICC) of this tool was also good (value for the whole tool was 0.95, and for the Factor 1, Factor 2 and Factor 3 were 0.95, 0.85 and 0.89, respectively).

## Discussion

The aim of this study was to examine the acceptability, reliability and construct validity of the Iranian version of the CBI among workers of different occupations. With regard to the importance of occupational burnout issue, which has many negative consequences for the families, society and organizations, more studies on this issue have important implications in terms of health and wellbeing as well as design and management of working systems. Nevertheless, this issue has not received adequate attention in Iran. This may be, partly, due to the lack of valid and reliable specific tools for measurement of occupational burnout in the country. This emphasizes the need for reliable and valid instru­ments to evaluate occupational burnout for Persian-speaking populations.

Internal consistency of the Persian CBI and also test-retest reliability of this tool were shown to be good. These findings are generally in agreement with the findings reported in previous research.^2,12,18^ The internal consistency reliability of the three factors of Persian CBI in our study using the Cronbach’s α was between 0.82 and 0.90, which is relatively similar to that reported by Mahmoudi et al (Cronbach’s α between 0.84 and 0.89) among nurses.^18^ Similarly, the ICC values in our study ranged between 0.85 and 0.95, which is similar to those reported by Mahmoudi et al (0.83–0.95).^18^ In addition to its good reliability, the results showed no floor or ceiling effect for the Persian CBI. This means that the Persian version of the CBI has no measuring limitation and reassures the practitioners of the validity of this instrument. Ceiling and floor effects of this instrument have not been explored in previous research, and therefore it is not possible to compare the results in this context. Additionally, the content validity of the Persian CBI was approved by both qualitative (using expert panel members’ feedback) and quantitative (agree­ment between expert panel members and acceptable CVR and CVI values) assessments. Again, this finding is in agreement with the results reported in some previous studies.^15,18^

Similar to finding of other studies, the three factors of the Persian CBI indicated good factor structure, suggesting that the three-factor model fit better than one- or two-factor model.^2,11-17^ This finding support differentiation of the three domains of this instrument. Nevertheless, our study showed a low factor loading for one of the items (WB_7_) on the work-related burnout scale. According to the EFA model, this item was omitted from the work-related burnout scale because it had no significant correlation with other items. Contrary to this finding, Mahmoudi et al^18^ considered four factors for this tool and divided the work-related burnout into two separate subscales. Although a low loading was found for this item, the authors did not remove this item and added it to the personal burnout.

The findings of this study demonstrated that the Iranian adaptation of the CBI is a reliable and valid instrument for measurement of burnout in Persian-language populations. These findings provide further evidence that the CBI can be used and applied in countries other than the origin country. In line with previous reports, the items of this instrument demonstrated a high degree of discrimination capacity and reliability (internal consistency and homogeneity).^10,14^

## Conclusion

This study was aimed to validate the Iranian version of CBI for the Iranian language (Persian) populations and the results indicated high degrees of reliabil­ity, feasibility, and validity for the Persian CBI as a tool for measurement of occupational burnout in workers of different occupations. The psy­chometric properties of the Persian version of the CBI and the original English version were consistent, which suggests that the Persian CBI can be used by Iranian research­ers and practitioners for evaluation of occupational burnout in different workplace settings and environments.

## Ethical approval

This study was approved by the ethics committee of the Tabriz University of Medical Sciences (IR.TBZMED.REC.1396.787).

## Competing interests

The authors declare that they have no competing interests.

## Disclaimer

The authors claim that no part of this manuscript has been copied from other sources.

## Authors’ contributions

EJ contributed to work design and data collection. ID contributed to the conception and work design as well as drafting the work. MAJ contributed to the analysis and interpretation of data.

## Acknowledgments

The authors would like to acknowledge all subjects who participated in this study.

**Table 1:** The scores of CVI and CVR of the Persian CBI

**Item**	**Item content**	**CVI**	**CVR**
Personal burnout			
1	How often do you feel tired?	0.97	1.00
2	How often are you physically exhausted?	1.00	1.00
3	How often are you emotionally exhausted?	1.00	1.00
4	How often do you think: “I can’t take it anymore”?	0.97	1.00
5	How often do you feel worn out?	0.93	1.00
6	How often do you feel weak and susceptible to illness?	1.00	1.00
Work-related burnout			
7	Is your work emotionally exhausting?	1.00	1.00
8	Do you feel burnt out because of your work?	1.00	1.00
9	Does your work frustrate you?	1.00	1.00
10	Do you feel worn out at the end of the working day?	1.00	1.00
11	Are you exhausted in the morning at the thought of another day at work?	0.97	1.00
12	Do you feel that every working hour is tiring for you?	0.97	1.00
13	Do you have enough energy for family and friends during leisure time?	1.00	1.00
Client burnout			
14	Do you find it hard to work with clients?	.091	1.00
15	Do you find it frustrating to work with clients?	1.00	1.00
16	Does it drain your energy to work with clients?	1.00	1.00
17	Do you feel that you give more than get back when you work with clients?	0.93	0.85
18	Are you tired working with clients?	1.00	1.00
19	Do you sometimes wonder how long you will be able to continue working with clients?	1.00	1.00

**Table 2:** Factors and factors loading for each test item^a^

**Item**	**Item description**	**Factor 1**	**Factor 2**	**Factor 3**
PB_1_	Feel tired	0.855		
PB_2_	Physically exhausted	0.829		
PB_6_	Feel weak and susceptible to illness	0.710		
PB_3_	Emotionally exhausted	0.699		
PB_5_	Feel worn out	0.685		
PB_4_	Can’t take it anymore	0.606		
CB_3_	Energy to work with clients		0.911	
CB_1_	Find it hard to work with clients		0.807	
CB_2_	Frustrating to work with clients		0.734	
CB_5_	Tired working with clients		0.689	
CB_4_	Give more than get back		0.360	
CB_6_	Able to continue working with clients		0.273	
WB_3_	Work frustrate			-0.901
WB_2_	Feel burnt out			-0.815
WB_4_	Feel worn out at the end of the work			-0.623
WB_1_	Emotionally exhausting			-0.600
WB_6_	Feel that every working hour is tiring			-0.536
WB_5_	Exhausted in the morning			-0..435

Extraction Method: Principal Factoring. Rotation Method: Oblimin with Kaiser Normalization. ^a^ Rotation converged in 8 iterations.

**Table 3:** Results of ceiling and floor effect

**Scale**	**Ceiling**	**Floor**
	**No. (%)**	**No. (%)**
Personal burnout	11 (1.5)	15 (2.0)
Work-related burnout	12 (1.6)	1 (0.1)
Client burnout	10 (1.3)	12 (1.6)
